# Caveats on the use of rotenone to estimate mixotrophic grazing in the oceans

**DOI:** 10.1038/s41598-020-60764-2

**Published:** 2020-03-03

**Authors:** Guilherme D. Ferreira, Albert Calbet

**Affiliations:** 1 Institut de Ciències del Mar, CSIC, Pg. Marítim de la Barceloneta, 37-49, 08003 Barcelona, Spain; 2 Marine Biological Section, University of Copenhagen, DK-3000 Helsingør, Denmark

**Keywords:** Microbial ecology, Ecophysiology

## Abstract

Phagotrophic mixotrophs (mixoplankton) are now widely recognised as important members of food webs, but their role in the functioning of food webs is not yet fully understood. This is due to the lack of a well-established technique to estimate mixotrophic grazing. An immediate step in this direction would be the development of a method that separates mixotrophic from heterotrophic grazing that can be routinely incorporated into the common techniques used to measure microplankton herbivory (e.g., the dilution technique). This idea was explored by the addition of rotenone, an inhibitor of the respiratory electron chain that has been widely used to selectively eliminate metazoans, both in the field and in the laboratory. Accordingly, rotenone was added to auto-, mixo-, and heterotrophic protist cultures in increasing concentrations (ca. 24 h). The results showed that mixotrophs survived better than heterotrophs at low concentrations of rotenone. Nevertheless, their predation was more affected, rendering rotenone unusable as a heterotrophic grazing deterrent. Additionally, it was found that rotenone had a differential effect depending on the growth phase of an autotrophic culture. Altogether, these results suggest that previous uses of rotenone in the field may have disrupted the planktonic food web.

## Introduction

The photosynthetic activity of marine phytoplankton is responsible for nearly half of the carbon (C) sequestration by autotrophs on Earth. Most of this C will be processed in the food web by microzooplankton^1,2^; however, it is currently accepted that a substantial part of this grazing activity might be mediated by phagotrophic “phytoplankton”^3^. Therefore, the flux of C throughout the food web becomes more complicated than predicted when considering only the phytoplankton-zooplankton dichotomy, as this type of plankton is concurrently a primary and a secondary producer^4^.

Mixotrophy is a globally ubiquitous nutritional strategy^5,6^ that defies the traditional auto- and heterotroph classification by combining both nutritional modes^7^ and can be found among phylogenetically diverse organisms whose sizes occupy four orders of magnitude^8^. Mixotrophy is traditionally defined as the use of both inorganic and organic C-forms but can also include the incorporation of other nutrients^7^. By definition, the passive uptake of dissolved organic C sources by some photoautotrophs such as diatoms^9^ can be regarded as mixotrophy, although this trait is not useful for discriminating among trophic strategies^4^. Thus, for the purpose of clarity, this paper will hereafter only address phago-mixotrophs, which have recently been termed mixoplankton^8^. Mixoplankton can be divided according to their physiological traits of chloroplast acquisition into two major groups, the Constitutive and the Non-Constitutive mixoplankton (CM and NCM, respectively)^10^. CMs possess an innate ability to photosynthesize, whereas NCMs acquire their C-fixation mechanisms from ingested prey.

The presence of mixoplankton, with this trophic mode being the rule rather than the exception^4^, impairs most of the current models for nutrient cycling dynamics^11^, fishery management^12^, and climate change predictions^13^. Indeed, mixoplankton may affect the stability of the food web^14^ while enhancing predators’ gross growth efficiencies and nutrient cycling^7,10^, which have consequences on models’ forecasting abilities. In addition, despite knowing that mixotrophy is abundant, it is still very difficult to quantify the degree of functional mixotrophy in a given microplankton assemblage^8^. Indeed, the traditional methods for estimating primary production in aquatic environments (such as ^14^C) do not disentangle the contribution of pure autotrophs and mixoplankton^8,15^. Similarly, the methods aiming at measuring the grazing and secondary production by phagotrophs fail, once again, to discriminate the contribution of mixoplankton^8^. It becomes thus clear that a better performance of ecosystem predictive models depends on a proper approach to the mixoplankton paradigm.

Therefore, to understand the contribution of mixoplankton to the trophic interactions of any given system, the quantification of auto- and heterotrophic processes is needed either at a community or individual level^15,16^. At the community level, the major issue regarding mixoplankton is the change in bulk nutrient circulation whereas at the individual level is to elucidate how mixoplankton affect the structure and biodiversity of a community^16^.

Several methodologies to measure mixotrophic grazing in the field do exist, in particular targeting bacterivorous nanoflagellates (reviewed recently in Wilken *et al*.^15^ and Beisner *et al*.^16^). The techniques with the highest success rate rely on fluorescent particles that are used as tracers (such as Fluorescently Labelled Bacteria^16^, and Live Fluorescently Labelled Algae^17^), especially because of the applicability to remote field locations^15^. These approaches have a high taxonomic resolution, both in terms of predators and prey. Additionally, due to the short-term nature of these experiments, it is easier to assess effects such as the relevance of diel feeding rhythms for a given organism/community^18^. Yet, as these methods rely on microscopy, a few disadvantages emerge and can restrict their application. The major disadvantage is the shortage of properly trained taxonomists who can readily identify mixoplankton within the samples^8^. Other important disadvantages include the lack of evidence of selectivity of grazers towards or against the labelled prey, the artificial increase in natural prey densities^17,19^, and the possible coincidental overlap of prey and predator cells upon filtration^15^.

An approach that does not possess any of the above mentioned disadvantages while retaining a short-term nature is the one that relies on the selective action of acidotropic probes (e.g. LysoTracker Green) on food vacuoles^20–22^. This fluorochrome can be coupled to flow cytometry, yielding a good discrimination of both mixo- and heterotrophic nanoflagellates communities^21^ and even estimates of *in situ* bacterivory rates by both groups^20^. Yet, it relies on the maintenance of the membrane potential, which is disrupted by fixation methods, restraining its use to live samples^22^. Additionally, the use of a flow cytometer is highly limited by the size of the particles, as larger and less abundant organisms such as dinoflagellates and ciliates (likely the major algal grazers) are mostly missed. Finally, unspecific fluorescence may occur on pigmented cells (e.g. fluorescence by silica frustule of diatoms^19^, acidic thylakoid lumens^15^ or autophagy of cellular components^23^).

Regarding community level approaches (which do not possess any of the above mentioned disadvantages but cannot provide differentiation between groups), the most widely used technique to measure microplankton herbivory in the field (the dilution technique^24^) is blind to mixotrophy (see Schmoker *et al*.^2^ for a review of the assumptions, details, and caveats of the methodology).

Should a natural sample contain mixoplankton (the most usual condition in marine waters), the mortality of prey measured on a standard dilution setting would not just be attributable to heterotrophic life-forms but also to mixoplanktonic organisms^25^. Additionally, knowing that both CMs and NCMs possess chlorophyll, mixoplankton act simultaneously both as prey and predators in the dilution technique, biasing the grazing estimates^2^.

It becomes thus evident that new approaches to estimate mixotrophic grazing *in situ* are required. Given that the dilution technique has proven to be a simple and useful technique but does not discriminate between mixo- and heterotrophic grazing, it would be very useful to develop a modified version of this technique that is capable of uncoupling the grazing rates for both groups. Therefore, a method that discriminates between trophic modes or one able to disrupt one of them would be extremely useful. In this regard, rotenone (IUPAC: (2 R,6aS,12aS)-1,2,6,6a,12,12a-hexahydro-2-isopropenyl-8,9-dimethoxychromeno[3,4-b]furo(2,3-h)chromen-6-one) is a compound that inhibits the electron transport chain in the mitochondria by blocking the transmission of electrons from complex I to ubiquinone^26^. Therefore, rotenone discontinues oxidative phosphorylation and ATP synthesis in this organelle. According to the mode of action, organisms relying exclusively on mitochondria for ATP synthesis (heterotrophs) are likely more vulnerable to rotenone than chloroplast-bearing organisms, which can also use chloroplasts to produce ATP in the light phase of the photosynthesis^27^.

Rotenone has already been suggested to eliminate unwanted predation by rotifers in microalgae cultures, as microalgae are seemingly unaffected^28–30^. Nevertheless, direct evidence of the effects of rotenone on chloroplast-bearing organisms is scarce despite the common assumption that these organisms are largely unaffected. If this assumption is confirmed, from a theoretical point of view, natural food webs could be modified by suppressing heterotrophic grazers^2^. It is important to mention that, also from a theoretical point of view, a dose of rotenone could diminish the pool of available ATP for chloroplast-bearing organisms as well, which ultimately may affect their grazing performance, both in the laboratory and in the field.

Therefore, the present study investigates the effects of rotenone on auto-, mixo- and heterotrophs in the laboratory under acute assays (ca. 24 h), using growth and ingestion as endpoints. The main aim of this study was to evaluate whether rotenone could be tentatively added to a standard dilution setting to uncouple mixo- and heterotrophic grazing rates. Furthermore, on a parallel and independent experiment, it was evaluated whether the physiological condition of an organism (assessed by a differential growth phase) affected its tolerance to rotenone.

## Results

### Rotenone effects on growth rates

**Figure 1:**
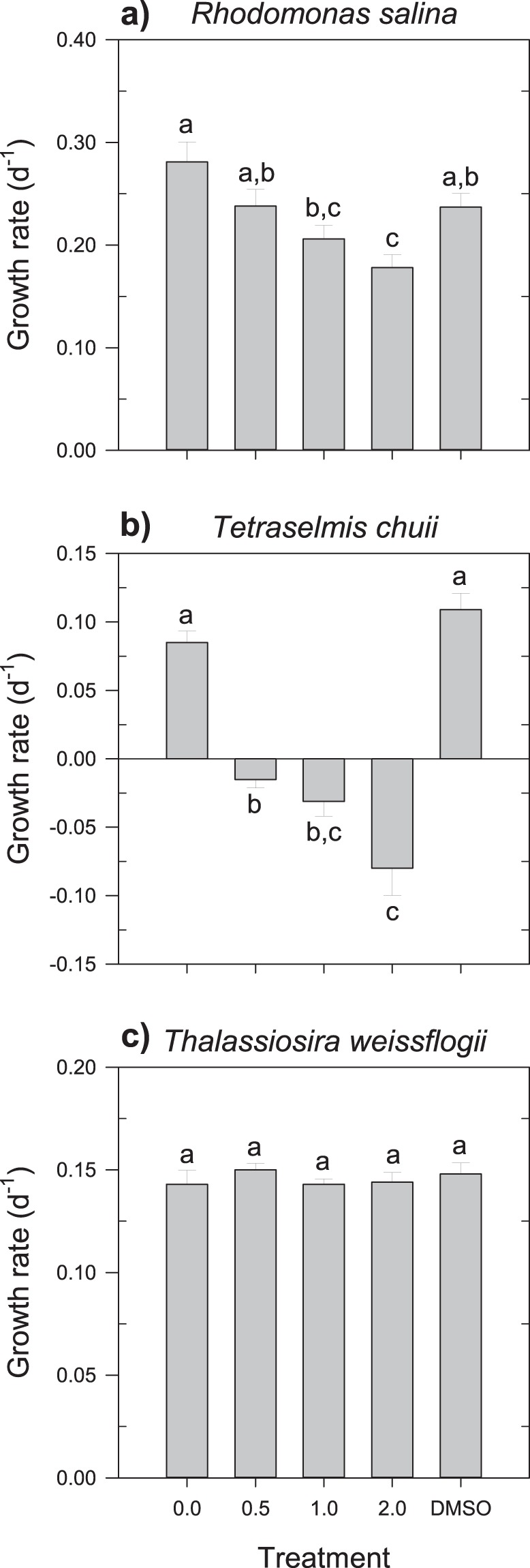
Growth rate (d^−1^) of the autotrophic (**a**) *R. salina*, (**b**) *T. chuii*, and (**c**) *T. weissflogii* upon exposure to increasing concentrations of rotenone. The data plotted for *R. salina* include all the results for the experiments with the different grazers. Different letters within the same organism indicate statistically significant differences (Tukey HSD, P < 0.05). Error bars ± s.e.m.

**Figure 2:**
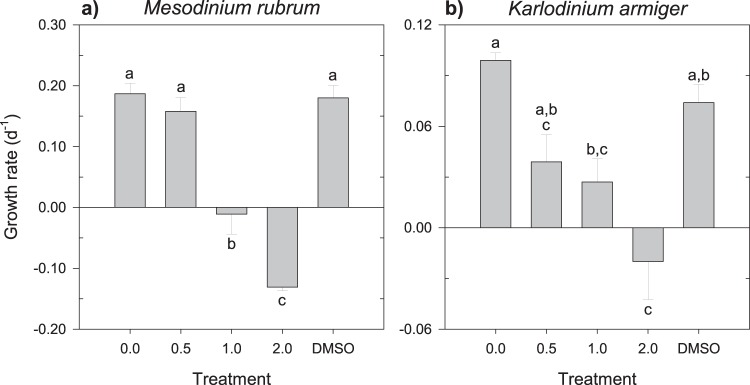
Growth rate (d^−1^) of the mixotrophic (**a**) *M. rubrum* and (**b**) *K. armiger* upon exposure to increasing concentrations of rotenone. Different letters within the same organism indicate statistically significant differences (Tukey HSD, P < 0.05). Error bars ± s.e.m.

The increase in rotenone concentration progressively reduced the growth rates of the two autotrophic flagellates tested (Fig. 1a,b). The response was more drastic in *Tetraselmis chuii*, which even displayed mortality at the lowest concentration (Fig. 1b). Conversely, at the same concentration, *Rhodomonas salina* was not significantly affected (Fig. 1a; Tukey HSD, P = 0.261). On the other hand, the diatom *Thalassiosira weissflogii* was unaffected by all concentrations of rotenone (one-way ANOVA, P = 0.792; Fig. 1c). DMSO at ca. 0.2% had no visible effect in any of the target autotrophic species when compared to the treatment with 0 mg L^−1^ (Tukey HSD tests, P > 0.05 in all cases). The mixoplankton *Mesodinium rubrum* and *Karlodinium armiger* were not significantly affected by the presence of DMSO or by the lowest concentration of rotenone, although a negative tendency was observed for *K. armiger* in this last instance (Fig. 2; Tukey HSD, P = 0.098). However, higher concentrations of the chemical compound severely reduced the growth rates of both protists, even resulting in mortality (Fig. 2).

**Figure 3:**
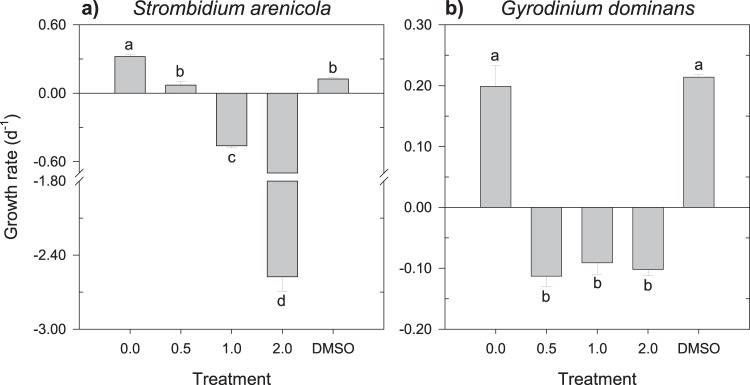
Growth rate (d^−1^) of the heterotrophic (**a**) *S. arenicola* and (**b**) *G. dominans* upon exposure to increasing concentrations of rotenone. Different letters within the same organism indicate statistically significant differences (**a** – Games-Howell, P < 0.05; **b** – Tukey HSD, P < 0.05). Error bars ± s.e.m.

The growth of the heterotrophic species was, on average, the most affected by rotenone (Fig. 3). For the heterotrophic dinoflagellate *Gyrodinium dominans*, the maximum growth inhibition was achieved immediately at the lowest concentration tested (0.5 mg L^−1^); at this concentration, however, the heterotrophic ciliate *Strombidium arenicola* still exhibited positive growth, although it was almost 80% lower than that under the control condition of 0 mg L^−1^. This ciliate species appeared to be particularly sensitive, being the only species significantly affected by the sole presence of DMSO in the water (Games-Howell, P = 0.017). Further supporting a high sensitivity of *S. arenicola*, the ciliates that remained alive after the 24 h were nearly immotile, suggesting severe deleterious effects. Independent of the trophic mode, all motile species exhibited a reduction in the speed of displacement in the presence of the highest concentrations of rotenone, although the magnitude of the reduction was not quantified.

### Rotenone effects on ingestion rates

**Figure 4:**
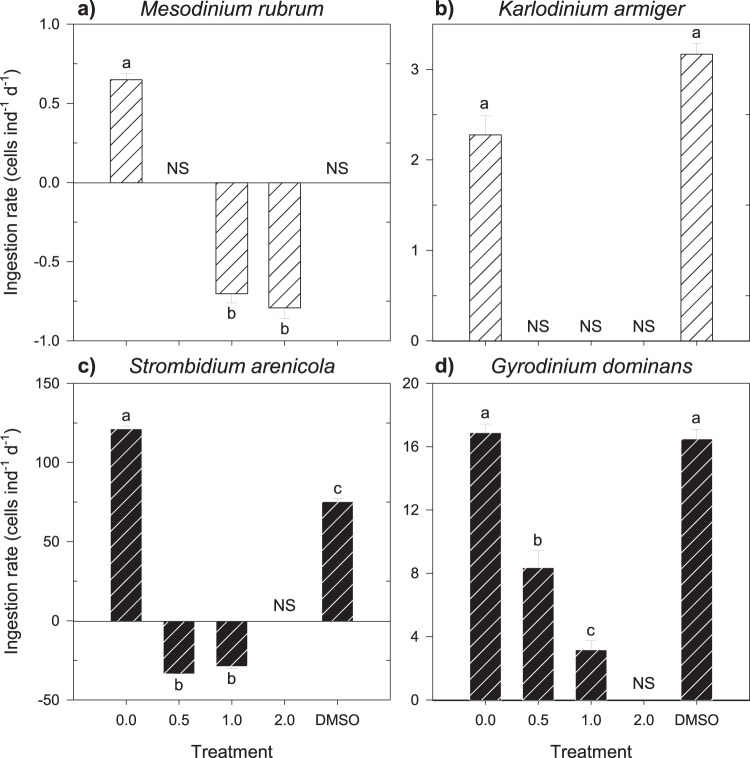
Ingestion rate (cells ind^−1^ d^−1^) of the mixotrophic () (**a**) *M. rubrum* and (**b**) *K. armiger*, and heterotrophic () (**c**) *S. arenicola* and (**d**) *G. dominans* upon exposure to increasing concentrations of rotenone. All organisms were fed *R. salina* during the exposure period. Different letters within the same organism indicate significant differences (Tukey HSD, P < 0.05). NS represents the non-significant ingestion rates (two-tailed Student’s t-test, P > 0.05). Error bars ± s.e.m.

The presence of rotenone impaired feeding in both ciliates, *M. rubrum* (Fig. 4a) and *S. arenicola* (Fig. 4c), regardless of the trophic mode of nutrition. The responses varied between no significant grazing (two-tailed Student’s t-tests, P > 0.05 in all cases) and significantly negative ingestion rates (Tukey HSD tests, P < 0.05 in all cases). A significantly negative ingestion rate (1.0 and 2.0 mg L^−1^ for *M. rubrum*, and 0.5 and 1.0 mg L^−1^ for *S. arenicola*) implies a positive growth of the prey in the experimental with respect to the control bottles, and likely results from an increase in the nutrient pool originating from the dead grazers. The presence of DMSO also deterred the feeding of these two species, with *M. rubrum* being the most affected (Fig. 4a,c). On the other hand, neither the mixotrophic *K. armiger* (Fig. 4b) nor the heterotrophic *G. dominans* (Fig. 4d) were significantly affected by the DMSO treatment (Tukey HSD tests, P > 0.05 in all cases). Rotenone, however, did affect the feeding rates of these dinoflagellates. *K. armiger* displayed no evidence of feeding whenever rotenone was present, and *G. dominans* showed null ingestion rates at 2.0 mg L^−1^ of rotenone (two-tailed Student’s t-tests, P > 0.05 in all cases).

### Rotenone effects on the overall grazing impact

The reduction in the impact of the chosen predators on the standing stock of *R. salina*, defined using Eq. (1) as the combined effect of feeding rates and grazer abundances during the incubations, is summarised in Table 1. Regardless of the trophic mode of nutrition, the grazing pressure by dinoflagellates was overall less inhibited by rotenone than that exhibited by ciliates.

**Table 1:** Combined effects of rotenone on grazer survival and on their feeding rates on *R. salina* (GIR, %) throughout the incubation.

Species (Trophic mode)	Grazing Impact Reduction (GIR), %				
	0.0 mg L^−1^	0.5 mg L^−1^	1.0 mg L^−1^	2.0 mg L^−1^	DMSO
*Gyrodinium dominans* (Heterotroph)	0.00	56.83	83.62	100.00^*^	0.15
*Strombidium arenicola* (Heterotroph)	0.00	123.07	114.80	100.00^*^	45.59
*Karlodinium armiger* (CM)	0.00	100.00^*^	100.00^*^	100.00^*^	0.00
*Mesodinium rubrum* (NCM)	0.00	100.00^*^	197.07	202.77	100.00^*^

The values were calculated using Eq. (1). Non-significant ingestion rates were considered 0, and GIR was thus capped at 100% in these situations, with the values highlighted with an *. No effect of the treatment on the overall grazing impact is indicated by a zero in the table.

### Effects of physiological conditions on the resistance to rotenone

**Figure 5:**
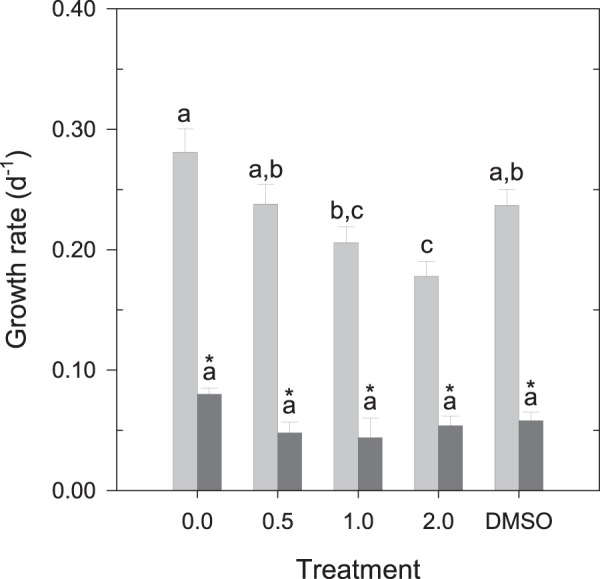
Growth rate (d^−1^) of the autotrophic *R. salina* upon exposure to increasing concentrations of rotenone on the exponential () and deceleration () growth phases. The data regarding the exponential phase is the same as that displayed in Fig. 1a. Different letters within the same growth phase indicate significant differences (Tukey HSD, P < 0.05). *Indicates statistically significant differences between exponential and deceleration phases for each individual treatment (Bonferroni, P < 0.05). Error bars ± s.e.m.

Rotenone affected *R. salina* in different ways depending on its physiological condition (Fig. 5). During the deceleration phase, the flagellate was roughly unaffected by the presence of rotenone, independent of the concentration (one-way ANOVA, P = 0.071). On the other hand, during exponential growth, progressively higher concentrations of rotenone diminished the growth of this cryptophyte up to a maximum of ca. 40% lower than that under the control treatment with 0 mg L^−1^ (Tukey HSD test, P = 0.000).

## Discussion

As expected, autotrophs were more resistant to rotenone, with the exception of *T. chuii*. Other species of the genus *Tetraselmis* are also reported to be more vulnerable to rotenone than other algae species, such as the marine *Nannochloropsis oculata*^29^ and the freshwater *Chlorella kessleri*^30^. This observation suggests that there may be a factor that is common to the genus *Tetraselmis* that enhances cellular susceptibility to this compound, although it is currently unknown. Conversely, the diatom and *R. salina* were quite resistant to rotenone effects. In the particular case of the cryptophyte, the effects of this compound were only evident during the exponential growth phase.

Cells undergo drastic metabolic changes when switching from exponential to stationary phases. For example, photosynthesis and respiration rates are, on average, higher during the exponential growth phase for auto- and mixotrophic species^31^. Similarly, heterotrophic ciliates and flagellates displayed higher respiration rates when actively growing than when stationary^32^. Therefore, it seems that the increased respiratory chain activity during the exponential phase enhances an organism’s susceptibility to rotenone. This conclusion aligns with the mechanism of action of rotenone, which, among other effects, is known to inhibit the synthesis of ATP^26^. A consequence of the reduced pool of available ATP can be seen in the assembly of microtubules, which becomes impaired and ultimately results in mitotic arrest and inhibition of cell proliferation^33^. Without these processes, the cell cannot divide, which would have a much higher impact on actively growing cells than on those progressing towards stationary phase.

The observed differences between growth phases under exposure to rotenone may have important consequences for the interpretation of laboratory and field experiments on single-celled organisms, not only with rotenone but with other toxic compounds as well. Despite having data exclusively from *R. salina* (which forces caution in the extrapolation of conclusions to other species), the results indicate that the effect of pollutants should always be tested using the same physiological conditions to minimise intra-specific differences. In the laboratory, this can be easily accomplished by controlling sampling times and/or by using a single batch of cultured organisms (as used in the experiments with mixo- and heterotrophs). On the other hand, for field work this may represent a challenge. Nonetheless, organisms in the field are likely living on an almost constant exponential growth phase (e.g. Dortch *et al*.^34^), diminishing the risk of comparisons.

A major result of the study is the high sensitivity of the heterotrophic ciliate *S. arenicola* to rotenone, in particular compared to the other heterotrophic predator tested, the dinoflagellate *G. dominans*. In fact, *G. dominans*, displayed a peculiar response to rotenone, showing negative growth rates and high ingestion rates at the lowest concentration, and similar growth rates and non-significant ingestion rates (two-tailed Student’s t-test, P = 0.114) at the highest. These results suggest that this dinoflagellate may be able to tolerate the presence of rotenone up to a concentration of 1.0 mg L^−1^ by maintaining key cellular processes (like phagocytosis) active while avoiding expensive ones such as cellular division. This could be a mechanism of survival that enables the endurance of harsh conditions for short time periods. Nonetheless, more data is needed to validate this hypothesis. On the other hand, planktonic ciliates are known to be highly susceptible to several chemical compounds, such as hydrocarbons and chemical dispersants^35,36^, but also to DMSO, although the toxicity of the latter is usually evidenced at higher concentrations than the ones used in this study^37,38^. Indeed, the ciliate *S. arenicola* was the only species whose growth was reduced by ca. 60% solely by the presence of DMSO.

Analogous to the results observed for heterotrophs, with the ciliate being more sensitive than the dinoflagellate, the mixotroph *M. rubrum* was more sensitive than *K. armiger*. In fact, the ingestion rate of *M. rubrum* was already negligible even with DMSO as the only added compound (Fig. 4a). Indeed, DMSO hindered the ingestion of prey for both ciliates (Fig. 4a,c). In this regard, a precursor of DMSO, β-dimethylsulfoniopropionate (DMSP), reduced the feeding of heterotrophic marine ciliates by 50–75%, whereas for heterotrophic dinoflagellates, this reduction was 28–40%^39^.

Overall, chloroplast-bearing predators displayed better resilience than heterotrophs at a concentration of 0.5 mg of rotenone L^−1^. However, their feeding rates were more affected, rendering the overall mixoplankton grazing impact on prey populations considerably lower than that of heterotrophic predators. In particular, *K. armiger* did not exhibit any evidence of feeding in the presence of rotenone (irrespective of concentration), while displaying only a slight negative growth rate in the highest concentration. These results are in agreement with those of previous studies on this species, in which it has been observed that *K. armiger* can survive long starvation periods using only chloroplasts for C acquisition, although barely dividing in the absence of food^40^, comparable to the non-significant ingestion rates observed in this study in the presence of rotenone.

One of the main motivations of this study was to test the effectiveness of the use of rotenone combined with the dilution technique to determine mixotrophic grazing. For the method to be useful, heterotrophic grazing should be impaired while leaving mixoplankton grazing unaffected. Despite the promising results for the effects of rotenone in terms of growth rates (with perhaps the caveat of the high sensitivity of *T. chuii*), the analysis of the effects of this compound on grazing highlighted severe limitations that were not predicted by the theoretical mechanism of action for rotenone (see Table 1). For instance, one of the assumptions of the study was that chloroplast-bearing organisms would be less affected by rotenone despite likely displaying a reduced ATP pool. A question that remains unanswered by this assumption is how large is the dependence of mixotrophic grazing processes on the ATP produced by the oxidative phosphorylation. In other words, can the photo phosphorylation supply enough ATP to maintain basal functions while enabling phagocytosis? The answer, at least from the present experiments, seems to be no, as explained next.

At 30 µE m^−2^ s^−1^ (the experimental conditions used in the present study), a well fed *K. armiger* fixes C at a higher rate than the maximum observed for unfed cells, and the chlorophyll *a* content is close to the maximum registered^40^. These observations suggest that this dinoflagellate maximises the use of the chloroplasts in situations akin to those used on the present study. Therefore, it seems plausible to assume that these conditions are less prone to magnify potential negative effects of rotenone on the behaviour of the CM.

For *M. rubrum*, it is known that the photosynthetic capacities depend on the quality of the chloroplasts acquired through the ingestion of cryptophytes from the genera *Teleaulax*, *Plagioselmis* or *Geminigera*^41^, and peak around 30 µE m^−2^ s^−1^ ^42^. Ultimately, the sequestered chloroplasts require the presence of active cryptophyte nuclei, and start to lose photosynthetic efficiency after 3 days without the adequate food source^43^. Accordingly, during the exposure to rotenone, the chloroplasts of the NCM were also likely close to their full potential despite being fed with *R. salina*.

Contrary to all other tested predators, the heterotroph *G. dominans* was still able to ingest *R. salina* under concentrations of rotenone up to 1.0 mg L^−1^ (although with ca. 9% mortality and a grazing impact reduction, GIR, of ca. 83%). Indeed, it is important to note that with a concentration of 0.5 mg L^−1^, the absolute number of *R. salina* cells ingested per *G. dominans* was approximately 3.8× higher than that of *K. armiger* and 13.2× higher than that of *M. rubrum* in their respective control situations.

Thus, despite the fact that the chloroplasts of both mixotrophs tested here should have been in good conditions and that the available ATP pool for *G. dominans* was (likely) severely reduced during the exposure to rotenone, field grazing estimates using rotenone would be, at best, conservative. Indeed, the analysis of the GIR suggests that in a hypothetical dilution setting with rotenone (0.5 mg L^−1^) and all 3 predators, *G. dominans* would still be the major grazer. Hence, mixotrophic grazing is clearly affected by the reduced ATP concentration, although future physiological studies are required to elucidate the actual contribution of the oxidative phosphorylation for mixoplankton and its role on phagotrophy.

An example that further corroborates that heterotrophic dinoflagellates can display a substantial grazing impact on natural populations (thus further complicating the use of a heterotrophic grazing deterrent such as rotenone) is the fact that some areas of the Mediterranean Sea possess a biomass of heterotrophic dinoflagellates approximately 4× higher than that of autotrophic (with high potential for mixotrophy^3^) and mixotrophic species combined (not in a bloom situation)^44^. Additionally, the average C-specific ingestion rate of heterotrophic dinoflagellates is ca. 4.5× higher than that of their mixoplankton counterparts (see Fig. 10 in Calbet *et al*.^45^ and references therein), meaning that one can assume that heterotrophs would impact prey nearly 20× more than mixoplankton. Assuming that *G. dominans* is a good representative of heterotrophic dinoflagellates^46^, the presence of 0.5 mg L^−1^ would still render their impact (see Table 1) on prey populations ca. 11× higher than that of mixoplankton, which would be virtually zero (as per *K. armiger* results). Thus, rotenone cannot be used as an addition to the standard dilution technique for the purpose of deterring heterotrophic predation.

On an ecological note, rotenone has been used for decades to kill undesirable fish species *in situ*, and typical concentrations varied between 0.5 and 5.0 mg L^−1^, depending on the sensitivity of the target species^47^. However, as evidenced by the results of this study, considerably lower concentrations cause nefarious or even lethal effects on several planktonic species of distinct taxonomic groups. Additionally, the half-life of rotenone in aquatic environments ranges from hours to weeks^48^ and depends on several factors, namely temperature and pH, increases in which quicken degradation^28^. Hence, this information, together with the data gathered in this study for protists, and previous studies on zooplankton^49,50^ and rotifers^29,30^ suggest that the indiscriminate use of this compound in the past may have had disastrous consequences for aquatic food webs, whose extent is largely unknown.

Despite the inability of acting as a deterrent of heterotrophic grazing, rotenone can still be used as a good algal crop protector, especially if the predator is a sensitive organism like a ciliate (present study) or a rotifer^29,30^. Nevertheless, future measures should always assess the effect of rotenone on the specific organism that is plaguing the algal culture, as differences in the sensitivity towards the compound are expected, as seen in the present study. Similarly, the sensitivity of the algal culture itself should also be acknowledged before the application of rotenone, and factors such as the growth phase may be exploited to minimise the nefarious effects in non-target organisms.

## Methods

### Cultures

The experiments were conducted with two heterotrophs, the dinoflagellate *Gyrodinium dominans* (strain ICM-ZOO-GD001) and the ciliate *Strombidium arenicola* (strain ICM-ZOO-SA001); two mixotrophs, the CM dinoflagellate *Karlodinium armiger* (strain ICM-ZOO-KA001) and the NCM ciliate *Mesodinium rubrum* (strain DK-2009); two autotrophic flagellates, *Rhodomonas salina* (strain K-0294) and *Tetraselmis chuii*, and one autotrophic diatom, *Thalassiosira weissflogii*.

*G. dominans*, *S. arenicola*, and *K. armiger* were kept at an irradiance of 15–55 μE m^−2^ s^−1^ in autoclaved 0.1 μm-filtered seawater that contained EDTA and trace metals in accordance with f/2 medium. *R. salina* was offered as prey to all three species *ad libitum*, being replenished upon depletion by the predator. *M. rubrum* was grown up to an optimal density under the same light conditions although kept in autoclaved 0.1 μm filtered seawater without the addition of metals. Moreover, since this ciliate can only grow on a specific cryptophyte clade^41^ and is highly sensitive to elevated prey concentrations, it was supplied with *Teleaulax amphioxeia* (strain K-1837) as prey during the scale-up procedure, maintaining a proportion of ca. 5 prey per predator, to avoid losing the culture due to a surplus of prey^51^.

*R. salina*, *T. amphioxeia*, and *T. chuii* were kept in f/2 medium using a chemostat approach, with fresh medium being added every second day. This approach ensures that the cultures are kept in exponential phase at any time. These organisms were irradiated at 100–200 μE m^−2^ s^−1^ provided by cool white fluorescent lights. *T. weissflogii* was grown under the same conditions with the exception that silicate was added to the medium and bubbling was applied to maintain cells in suspension. All cultures were kept in a controlled-temperature room at 19 °C with a 10:14 L/D cycle. Additionally, all cultures were maintained at a salinity of 38 psu.

### Acute toxicity assays with rotenone

Rotenone solutions were prepared according to the guidelines provided by El-Sayed *et al.*^28^. Briefly, a stock solution of 1 g L^−1^ (2.535 mM) was prepared by dissolving 0.05 g of rotenone (≥ 95%, Sigma-Aldrich) in 50 mL of dimethylsulfoxide (DMSO) (≥ 99.5%, PanReac AppliChem) and stored at −20 °C while not in use. Standard solutions of 50 mg L^−1^ were prepared on the days of the experiments by diluting the stock solution 20× with deionized water.

The experiments were conducted in 132 mL Pyrex bottles, out of which 126.7 mL were plankton growing medium (see details below), and the remaining 5.3 mL contained varying proportions of the standard rotenone solution (50 mg L^−1^) and deionized water, to achieve the desired target concentration. The experiments consisted of three concentrations of rotenone (0.5, 1.0 and 2.0 mg L^−1^, i.e., 1.27 to 5.08 μM), and two controls, one containing only 5.3 mL of deionized water (0 mg L^−1^) and another with 5.3 mL of DMSO (solvent control). DMSO controls comprised only the highest concentration of DMSO (ca. 0.2%) used with the rotenone solutions of 2.0 mg L^−1^. The organisms were exposed to all concentrations simultaneously, and the growth and ingestion rates measured for each concentration.

The plankton growing medium used on experimental (predator and prey) and control (only prey) suspensions for all predators was prepared with the addition of 50 mL of fresh f/2 medium per L of suspension (final nutrient concentration equivalent to f/40 medium). The objective was to avoid nutrient limitation for the prey during the exposure period. These suspensions were then used to fill triplicate experimental and control bottles. Aside from these bottles, another experimental and control bottles were also filled at the same time using the same suspensions, to measure the initial concentration of the organisms^45,52^. All bottles were filled progressively, in three to four steps. Rotenone was added to the bottles with an automatic pipette just before capping them, to avoid exposing the organisms to very high concentrations of the compound, although only temporarily. The suspension was gently stirred between fillings. The formation of air bubbles during the filling and capping processes was avoided because shear may damage the organisms^53^ and thus bias the measured rates, also justifying the use of independent bottles for the initial measurements.

The experimental and control bottles were mounted on a plankton wheel (0.2 rpm) and incubated for 24±2 h at 19 °C with a 10:14 L/D cycle at ca. 30 μE m^−2^ s^−1^ during the light period. The treatments were sampled from the highest to the lowest concentration to compensate for the difference in the sampling times despite including that difference in the calculations for both vital rates. The incubations begun shortly after the onset of the light period to maximise the odds of survival for chloroplast-bearing organisms by enabling a longer period for ATP synthesis using the light phase of the photosynthesis^28,48^.

During the exposure period, all predators were given *R. salina* as prey at saturating concentrations (Table 2), to minimise the effect of different food concentrations on the measured ingestion rates. *M. rubrum* was acclimated to *R. salina* as prey for two days before the exposure to rotenone (after depleting *T. amphioxeia* from the culture medium). *M. rubrum* feeds on *R. salina*^41^ and relies on previously acquired chloroplasts^51^ for phototrophy, making *R. salina* a suitable prey to evaluate whether feeding still occurred in the presence of rotenone. Predator concentrations were adjusted to allow for ca. 30% prey depletion after the incubation time^52^. All organisms were counted and their volumes assessed using a Beckman Coulter Multisizer III particle counter, with the exception of *M. rubrum*, which can escape the current flow generated by the particle counter due to their shear sensitivity^54^. Aliquots of the experiments with *M. rubrum* were therefore fixed in acidic Lugol’s (final concentration 5%) and enumerated manually using a Sedgwick-Rafter counting chamber. A minimum of 300 cells (of both predator and prey) were counted using a 10× objective on an inverted microscope.

**Table 2:** Summary of the prey and predator concentrations used for the acute toxicity assays with rotenone for the mixotrophic *M. rubrum* and *K. armiger*, and heterotrophic *S. arenicola.* and *G. dominans*.

Species	Target concentration, Cells mL^−1^		Reference
	Prey	Predator	
*Gyrodinium dominans*	100 000	1 500	Calbet *et al*. ^52^
*Mesodinium rubrum*	15 000	1 500	Smith & Hansen^51^
*Karlodinium armiger*	100 000	3 750	Arias, unpublished
*Strombidium arenicola*	100 000	400	Arias, unpublished

### Growth and grazing impact under exposure to rotenone

Grazing rates and average prey concentrations were calculated after ca. 24 h using Frost^55^ equations; the average concentration of grazers in each replicate was used to assess the grazing per predator^56^. The magnitude of the effects of the different concentrations of rotenone on the GIR (%) on the prey populations was assessed separately for each grazer species by Eq. (1)

1 $$GI{R}_{speciesA}=100-(\frac{ < C{ > }_{i}\times {I}_{i}}{ < C{ > }_{0}\times {I}_{0}}\times 100)$$

where <*C*> and *I* are the mean predator concentration (cells mL^−1^) during the incubation period and the average ingestion rate (cells ind^−1^ d^−1^) measured for each treatment *i* (<*C*>_0_ and *I*_0_ refer to the control treatment of 0 mg L^−1^). The control suspensions with only DMSO added (no rotenone) were considered a treatment. Non-significant ingestion rates (see section Statistical analysis) were considered 0 for the calculation of the GIR.

### Physiological condition effects on the response to rotenone

It was additionally assessed whether the physiological condition of an organism affected its response to rotenone, as an increased respiratory chain activity during the exponential phase^31,32^ may enhance an organism’s susceptibility to the pollutant. *R. salina* was exposed to rotenone both during the exponential and deceleration growth phases, with all target concentrations tested at once. The experiments were conducted with this cryptophyte due to the simplicity of sampling different growth phases in a chemostat. Indeed, to sample the deceleration phase for the experiment, *R. salina* was simply allowed to grow for three more days in the chemostat without refreshing the f/2 medium. The experimental protocol was the same as described above.

### Statistical analysis

Species-specific effects of rotenone on growth were analysed using one-way ANOVAs followed by Tukey HSD post hoc tests (n = 15 for each treatment)^57^. In the case of *S. arenicola*, in which the assumptions of homoscedasticity were not met, the non-parametric Kruskal-Wallis test was applied, followed by the Games-Howell post hoc test (n = 15)^57^.

Ingestion rates were deemed significant only when the prey growth rates in the control and experimental bottles were significantly different (two-tailed Student’s t-test, n = 6 for each treatment)^58^. Subsequently, the results for this parameter were analysed with the same procedure as described for growth rates, with the normality and homoscedasticity assumptions met and Tukey HSD post hoc tests applied^57^. Finally, the effects of rotenone on the exponential and decelerating *R. salina* were analysed separately for each growth phase using one-way ANOVAs followed by Tukey HSD post hoc tests. The interaction between growth phases and treatment was assessed using a two-way ANOVA followed by Bonferroni post hoc tests due to the unequal sample size between factors^57^. Calculations were conducted using IBM SPSS Statistics 25, and all the results were considered significant at P < 0.05. All figures were generated using the software SigmaPlot 14.0.
